# Commentary: Mental Toughness and Individual Differences in Learning, Educational and Work Performance, Psychological Well-being, and Personality: A Systematic Review

**DOI:** 10.3389/fpsyg.2017.02329

**Published:** 2018-01-09

**Authors:** Daniel F. Gucciardi

**Affiliations:** School of Physiotherapy and Exercise Science, Curtin University, Perth, WA, Australia

**Keywords:** mentally tough, psychometrics, factorial validity, methodological quality, construct validity

The concept of mental toughness (MT) has garnered substantial interest over the past two decades. Scholars have published several narrative reviews of this literature (e.g., Connaughton et al., 2008; Crust, 2008; Gucciardi, 2017), yet in ~30 years of research there has been no attempt to review this body of work systematically to understand how MT is associated with hypothesized correlates. The systematic review by Lin et al. (2017) was timely for the field of MT (see also, Cowden, 2017). However, in this commentary, I explain two reasons why the conclusions drawn from this systematic review may be misleading or premature.

## Methodological quality of primary studies matters

Well-executed systematic reviews offer many advantages for summarizing, synthesizing and integrating findings across studies when compared with non-systematic evaluations (e.g., clear and accountable methods; see Gough et al., 2017). However, the potential value of even the most well-executed systematic review could be undermined by the methodological quality of the primary studies (Moher et al., 2015; Shamseer et al., 2015; Oliveras et al., 2017). An assessment of methodological quality is necessary both for determining what primary studies might be included in a systematic review and for making inferences regarding the reliability of those studies retained for analysis and integration. For example, differences in the methodological quality of primary studies might explain why research on the same topic results in different or conflicting degrees of evidence. The exclusion of a formal assessment of methodological quality is a major limitation of the systematic review conducted by Lin et al. (2017) because any bias in primary studies transfers to the synthesized evidence unless those biases and sources are variation are handled as part of the analysis and interpretation of the cumulative findings. The issue of statistical power is an important consideration in this regard, yet sample size justification is an often overlooked consideration among primary research on MT including my past work (e.g., Gucciardi and Jones, 2012). For example, should a study with 16 participants (90% power to detect the smallest possible effect of *r* = 0.67 at *p* < 0.05; Cowden et al., 2014) be given the same degree of quality as one with 351 participants (90% power to detect the smallest possible effect of *r* = 0.171 at *p* < 0.05; Cowden et al., 2016)? Although the answer depends on the smallest effect size of interest (Lakens and Evers, 2014), it is important to bear in mind that underpowered studies inflate false positives (Button et al., 2013) and effect sizes tend to be unstable when samples are small (Schönbrodt and Perugini, 2013). The process of assessing methodological quality across a heterogeneous set of primary studies is challenging, particularly for observational research (Vandenbroucke et al., 2014; von Elm et al., 2014), because of the unavailability of consensus regarding definitions, assessments, and integration with the synthesis of evidence (Oliveras et al., 2017).

## Construct validity evidence of psychometric tools matters

Given the predominance of self-reported MT among the primary studies of Lin et al.'s (2017) systematic review, a key consideration for the assessment of methodological quality is the degree of construct validity evidence for each tool. Construct validation refers to the testing of bidirectional associations between theory development and measurement in terms of assessments of the theoretical domain and its operationalization (substantive phase), empirical fidelity of the measurement approach (structural phase), and the meaning of test scores with key correlates or group differentiation (external phase) (Loevinger, 1957). Assuming sufficient evidence exists for the substantive foundations of a measure (e.g., precise definition, content validity evidence), ongoing tests of the internal structure of a scale are a necessary prerequisite for examinations of external relations because the number of latent factors or loading patterns may differ across samples, populations, and settings (Flora and Flake, 2017).

Lin et al.'s (2017) findings showed that the MTQ48 and its shortened version (MTQ18) (Clough et al., 2002) are the most widely used measures for the assessment of MT and its associations with external variables including cognition and educational, work and military performance (11 of 16 studies), psychological well-being (17 of 23 studies), personality and other psychological traits (9 of 16 studies) and genetics (4 of 5 studies). Yet psychometric analyses of the MTQ48 by the original author and his colleagues (Clough et al., 2012; Gerber et al., 2013; Perry et al., 2013, 2015) as well as independent researchers (Gucciardi et al., 2012, 2013; Birch et al., 2017; Vaughan et al., 2017) cast doubts on the operationalization of the 4Cs model of MT via the MTQ48 both in terms of global (i.e., model-data congruence) and local (i.e., pattern of factor loadings) misfit. As such, any conclusions regarding the associations between MT and key correlates are tenuous because of the uncertainty regarding the meaning of the underlying latent factor.

## Conclusion

As the first systematic review of the quantitative literature on the associations between MT and key correlates, I commend Lin et al. (2017) for their efforts in bringing together disparate literatures. However, I urge caution to those readers who might interpret their findings uncritically in two key ways. The exclusion of an assessment of the methodological quality of primary studies and the reliance in the literature on a measure of MT with questionable conceptual underpinnings and limited construct validity evidence reduce our confidence in the veracity of the available findings and, therefore, the conclusions and implications of the systematic review for theory and practice. An assessment of the methodological quality of primary studies included within the Lin et al. review (e.g., Mixed Methods Appraisal Tool; Pluye and Hong, 2014) and re-analysis and re-interpretation of the findings represents an important step for the science of MT. Indeed, a critical analysis of the methodological quality of primary work alone can represent a major contribution to a field of research because it might highlight deficiencies and/or strengths in evidence (Moher et al., 2015; Shamseer et al., 2015).

## Author contributions

The author confirms being the sole contributor of this work and approved it for publication.

### Conflict of interest statement

The author declares that the research was conducted in the absence of any commercial or financial relationships that could be construed as a potential conflict of interest.
